# Evidence‐based region of interest (ROI) definition for surface‐guided radiotherapy (SGRT) of abdominal cancers using deep‐inspiration breath‐hold (DIBH)

**DOI:** 10.1002/acm2.13748

**Published:** 2022-08-10

**Authors:** Yulin Song, Xingchen Zhai, Yubei Liang, Chuan Zeng, Boris Mueller, Guang Li

**Affiliations:** ^1^ Department of Medical Physics Memorial Sloan Kettering Cancer Center New York New York USA

**Keywords:** breast cancer, deep‐inspiration breath‐hold (DIBH), optical surface imaging (OSI), surface‐guided radiotherapy, treatment procedure

## Abstract

To define and evaluate the appropriate abdominal region of interest (ROI) as a surrogate of diaphragm positioning in deep‐inspiration breath‐hold (DIBH) for surface‐guided radiotherapy (SGRT) of abdominal cancers using 3D optical surface imaging (OSI). Six potential abdominal ROIs were evaluated to calculate their correlations with the diaphragm position using 4DCT images of 20 abdominal patients. Twelve points of interest (POIs) were defined (six on the central soft tissue and six on the bilateral ribs) at three superior–inferior levels, and different sub‐groups represented different ROIs. ROI‐1 was the largest, containing all 12 POIs from the xiphoid to the umbilicus and between the lateral body midlines while ROI‐2 had only eight inferior POIs, ROI‐3 had six lateral POIs, and ROI‐4 had four superior‐lateral POIs over the ribs, ROI‐5 contained six central and two most inferior‐lateral POIs and ROI‐6 contained six central and four inferior‐lateral POIs. Internally, the right diaphragm dome was used to represent its positions in 4DCT (0% and 50% within the cycle). The Pearson correlation coefficients were calculated between the diaphragm dome and all 12 external POIs individually or grouped as six ROIs. The quality of the abdominal ROIs was evaluated as potential internal surrogates and, therefore, potential ROIs for SGRT DIBH setup. The four most inferior POIs show the highest mean correlation (*r* = 0.75) with diaphragmatic motion, and the correlation decreases as POIs move superiorly. The mean correlations are the highest for ROIs with little or no rib support: *r* = 0.67 for ROI‐2, *r* = 0.64 for ROI‐5, and *r* = 0.63 for ROI‐6, while lower for ROIs with rib support: ROI‐1 has *r* = 0.60, ROI‐3 has *r* = 0.50, and ROI‐4 has only *r* = 0.28. This study demonstrates that the rectangular/triangular soft‐tissue ROI (with little rib support) is an optimal surrogate for body positioning and diaphragmatic motion, even when treating tumors under the rib cage. This evidence‐based ROI definition should be utilized when treating abdominal cancers with free‐breathing (FB) and/or DIBH setup.

## 1 INTRODUCTION

Surface‐guided radiotherapy (SGRT) using 3D optical surface imaging (OSI) has been widely used in radiotherapy clinics to provide real‐time guidance for patient setup and motion monitoring during treatment, such as intra‐cranial stereotactic radiosurgery and breast deep‐inspiration breath‐hold (DIBH).^1^, ^2^ In recent years, a trend of using OSI to replace conventional maker‐based techniques, such as the respiratory positioning management (RPM) system or marker fiducials, has appeared to provide faster and more accurate clinical procedures^3^, ^4^, ^5^ with improved patient safety.^6^ SGRT offers many advantages over the marker‐based techniques, including eliminating marker placement uncertainty, providing a very large field of view for patient setup, minimizing tissue deformation by aligning both the treatment site and associate anatomy that affects the site, such as the arm/chin in breast setup, and monitoring external motion with a comprehensive view.^7^ A task group (TG‐302) of the American Association of Physicists in Medicine has just published the SGRT guideline.^8^ Clinically, SGRT may replace the tattoo‐laser setup method, which has been established since the beginning of radiotherapy, providing improved patient care.^9^, ^10^, ^11^, ^12^ Although the body surface may contain both rigid and deformable skin surfaces, relatively reliable surfaces have been investigated to serve as a region of interest (ROI) for each disease site, including the breast, brain, and head and neck cancers. It is critical to find reliable ROIs on the skin surfaces to serve as a good body surrogate for patient setup.^13^, ^14^, ^15^

It is worthwhile to mention that a good body surrogate may not be an optimal tumor surrogate. Therefore, SGRT can replace skin tattoos but may never replace in‐room radiographical or magnetic resonance imaging (MRI) to localize a tumor in image‐GRT (IGRT). The only exception is that a surface ROI can also serve as a tumor surrogate, and an appropriate safety margin is applied based on SGRT setup uncertainty, such as facial ROI for conventional brain radiotherapy (a deep‐seated lesion in rigid anatomy)^11^ and breast ROI for conventional whole breast treatment (a superficial lesion).^16^ For SGRT breast setup, regardless of free‐breathing (FB) or DIBH, the same breast ROI can be used as the surrogate for both body position and DIBH chest elevation in the anterior‐posterior (AP) direction.^17^

Various ROIs have been suggested and used clinically for abdominal patient setup, including the central and/or lateral regions.^18^, ^19^ To anchor the body position, the anterior surface above lateral body midlines in the upper abdomen can serve as a good ROI, especially in the area with bony support, as the surface anatomy above the rib cage moves less than the soft abdominal surface. This ROI provides a uniquely identifiable landscape to align the on‐site OSI image to the external body contour in the planning computed tomography (CT) image in digital image communication in medicine format (DICOM). However, to infer the respiratory motion of the diaphragm, the stable rib cage ROI may not be suitable.^19^, ^20^, ^21^ From FB to DIBH setup, whether the same ROI would apply is a question we need to address to guide clinical application, as the alignment target changes from the external to internal.^18^, ^22^ Clinically, an RPM‐based indicator has also been studied and utilized for abdominal DIBH treatment,^23^ and the diaphragm has been used as a surrogate of tumor motion for respiratory‐gated ratiotherapy.^24^, ^25^

In this study, we systematically simulated and evaluated six different ROIs for the abdominal DIBH SGRT setup, including two rectangular ROIs, two lateral rib‐cage ROIs, and two central soft‐tissue triangular ROIs. Twenty abdominal patients’ clinical 4DCT images were used to quantify external surface and internal organ motion. The primary objective was to evaluate whether the ROI could be a surrogate for both body alignment and internal diaphragm motion. To simplify the study, 12 points of interest (POIs) in the abdominal region were defined to represent different ROIs by different grouping, and full inhalation (0%) and complete exhalation (50%) 4DCT images were examined to identify the positions of the diaphragm in the two extreme phases. The external–internal correlations of both individual and grouped POIs (ROIs) were analyzed and used to assess the validity of the six ROIs.

## 2 METHODS

### 2.1 Patient internal and external image data and six surface ROIs

Twenty abdominal patients’ clinical 4DCT images were used retrospectively to study ROIs for abdominal SGRT DIBH patient setup; and both external body surface and diaphragmatic motion were manually measured and quantitatively analyzed. Therefore, the feasibility of using the ROIs as a potential surrogate of respiratory motion was assessed to infer the diaphragm position for the DIBH setup.

Six potential ROIs were defined and analyzed. First, a large rectangular upper‐abdominal ROI‐1 was defined as the largest ROI from the xiphoid process to the biblical point in the SI direction and between the body midlines on the lateral sides. ROI‐2, covered the inferior half of the ROI‐1. Second, two rib‐cage ROIs (ROI‐3 and ROI‐4) were defined on both lateral body sides with the rib support with various inferior coverages. Third, two triangular soft abdominal ROIs (ROI‐5 and ROI‐6) were defined to contain the central soft tissue and lateral‐inferior body surface.

### 2.2 Twelve POIs and different sub‐groups to represent ROIs

**FIGURE 1:**
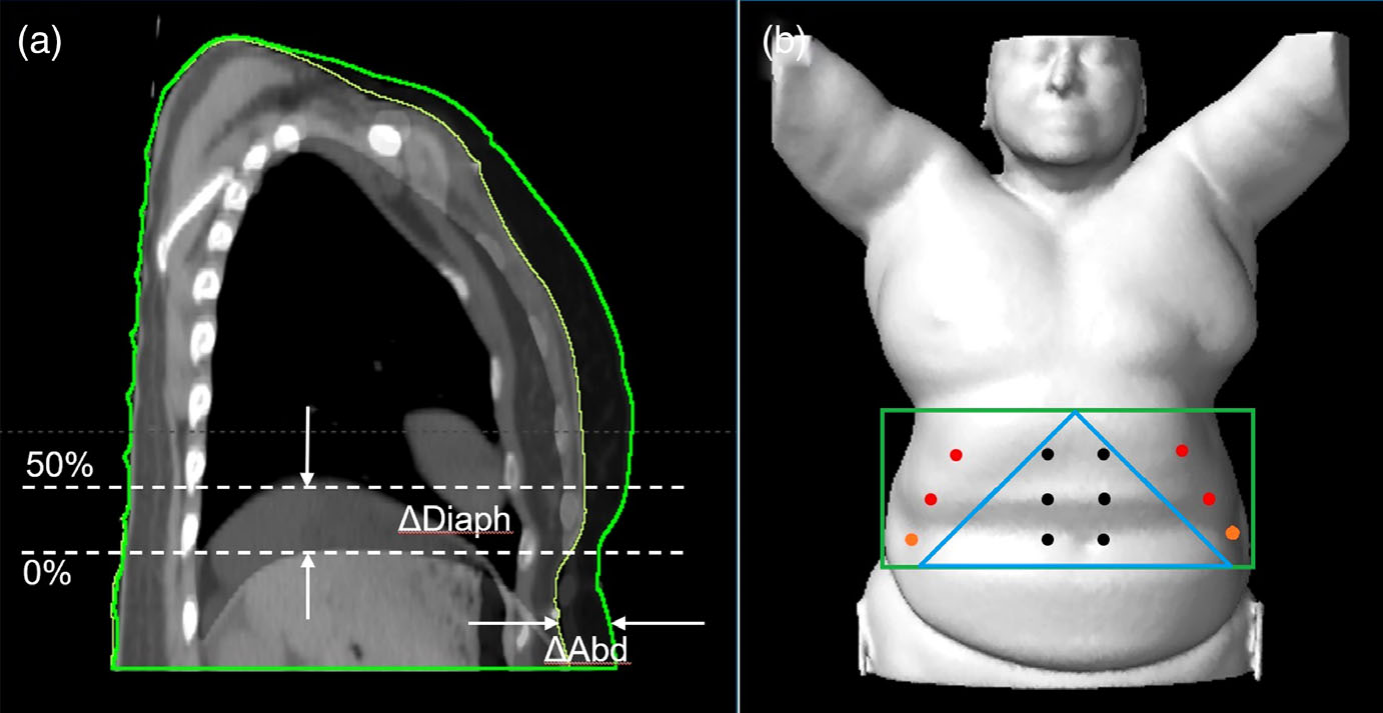
Illustration of the measurement of the diaphragmatic motion between inhalation (0%) and exhalation (50%) 4DCT images, the definition of six regions of interest (ROIs), and the selection of the 12 points of interest (POIs) in the abdominal region. (a) measurement of right diaphragm apex motion and (b) definition of 12 POIs at three longitudinal levels and on ribs (red) and soft tissue (black). The six ROIs are defined as: (1) ROI‐1: rectangular green box with all 12 POIs and ROI‐2: eight inferior POIs, (2) ROI‐3 and ROI‐4: lateral 4 (red) and six POIs (red and orange), respectively, and (3) ROI‐5 and ROI‐6: six POIs (black) in the blue triangle with two and four inferior‐lateral POIs, respectively

To simplify the ROI evaluation, 12 POIs were selected and combined differently to represent the six ROIs, as shown in Figure 1. The 12 POIs were placed in three position levels in the SI direction, and at each level, two were placed symmetrically at the body midline, and two were placed laterally on or near the rib cage edges. All 12 POIs represented rectangular ROI‐1, and eight inferior POIs represented ROI‐2. The six lateral and the four superior‐lateral POIs represented ROI‐3 and ROI‐4, respectively. The six central POIs plus two or four most inferior POIs represented the triangular soft tissue ROI‐5 or ROI‐6, respectively. To assess the external and internal motion relationship, only full inhalation (0%) and exhalation (50%) images within the 4DCT were used. Motions were measured on the individual POIs in the AP direction and the right diaphragm apex in the SI direction. In this manner, we could evaluate which of the six ROIs would be the best surrogate for both diaphragm motion and body position for abdominal DIBH setup.

### 2.3 Correlation between the 12 POIs and three ROIs with the diaphragmatic motion

To evaluate which ROI has the higher external–internal correlation, the 12 individual and grouped POIs were used to calculate the Pearson correlation coefficients:

(1) $$r\;=\frac{\sum \;\left(x_{i}-\bar{x}\right)\;\left(y_{i}-\bar{y}\right)}{\sqrt{\sum {\left(x_{i}-\bar{x}\right)}^{2}\sum {\left(y_{i}-\bar{y}\right)}^{2}}}\;,$$

where *x_i_* is the internal motion at the right diaphragmatic apex, and y_i_ is the external motion of a particular patient at an individual or grouped POIs, while $\bar{x}$ and $\bar{y}$ are their means of all 20 patients, respectively.

The correlation for each of the 6 ROIs was calculated using the diaphragmatic motion as the independent variable and the average of the individual POIs within the ROI as the dependent variable. These correlations were used to assess the validity of the ROIs as a surrogate of the diaphragmatic motion.

## 3 RESULTS

### 3.1 Correlation coefficients of individual 12 POIs with the diaphragmatic motion

**FIGURE 2:**
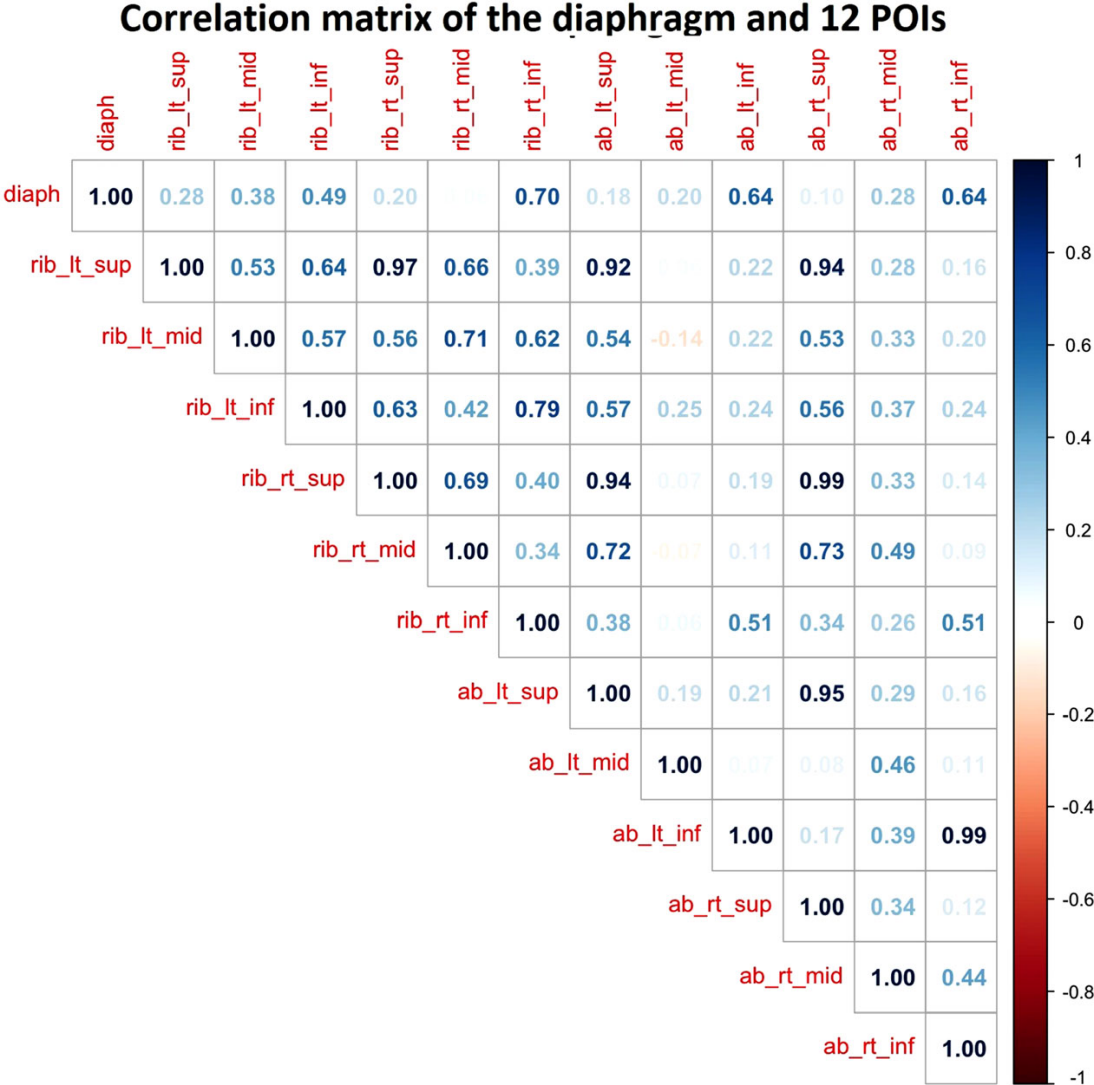
Pearson correlation matrix for all 12 points of interest (POIs) and the diaphragmatic apex among 20 abdominal patients. The four most inferior POIs show the highest correlations and the correlation decreases as the positions of the POIs move superiorly. The four most superior POIs illustrate the strongest correlation with each other. The POIs on the mid and inferior abdominal (ab) soft tissue and on the ribs do not correlate well, suggesting very different motion behaviors

**FIGURE 3:**
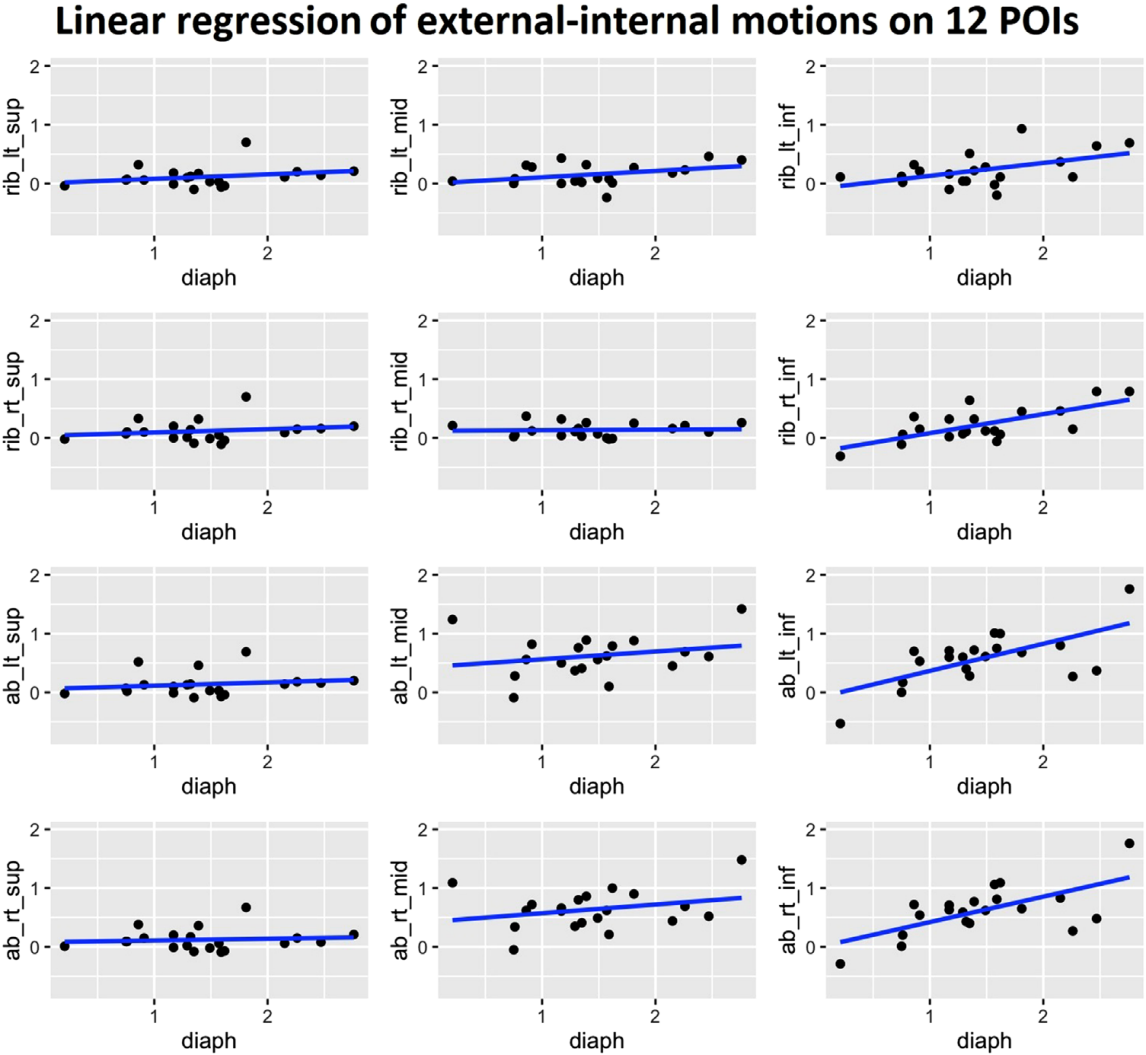
Linear regression fitting between the external (y‐axis) and internal (x‐axis) motions over the 12 POIs among 20 abdominal patients based on the measurements on 4DCT images. The slopes for the four most inferior POIs are the highest

Figure 2 illustrates the correlation coefficients of external‐internal motion for all 12 POIs and the right diaphragmatic apex. The correlations are the highest (*r* = 0.7) for the four most inferior POIs and decrease as the POIs move superiorly. The four most superior POIs have strong correlations (*r* > 0.9) with each other. When performing linear regression fitting, the four most inferior POIs illustrate distinguished slopes from other POIs, as shown in Figure 3.

### 3.2 The average correlation of six grouped POIs as ROI representatives

**FIGURE 4:**
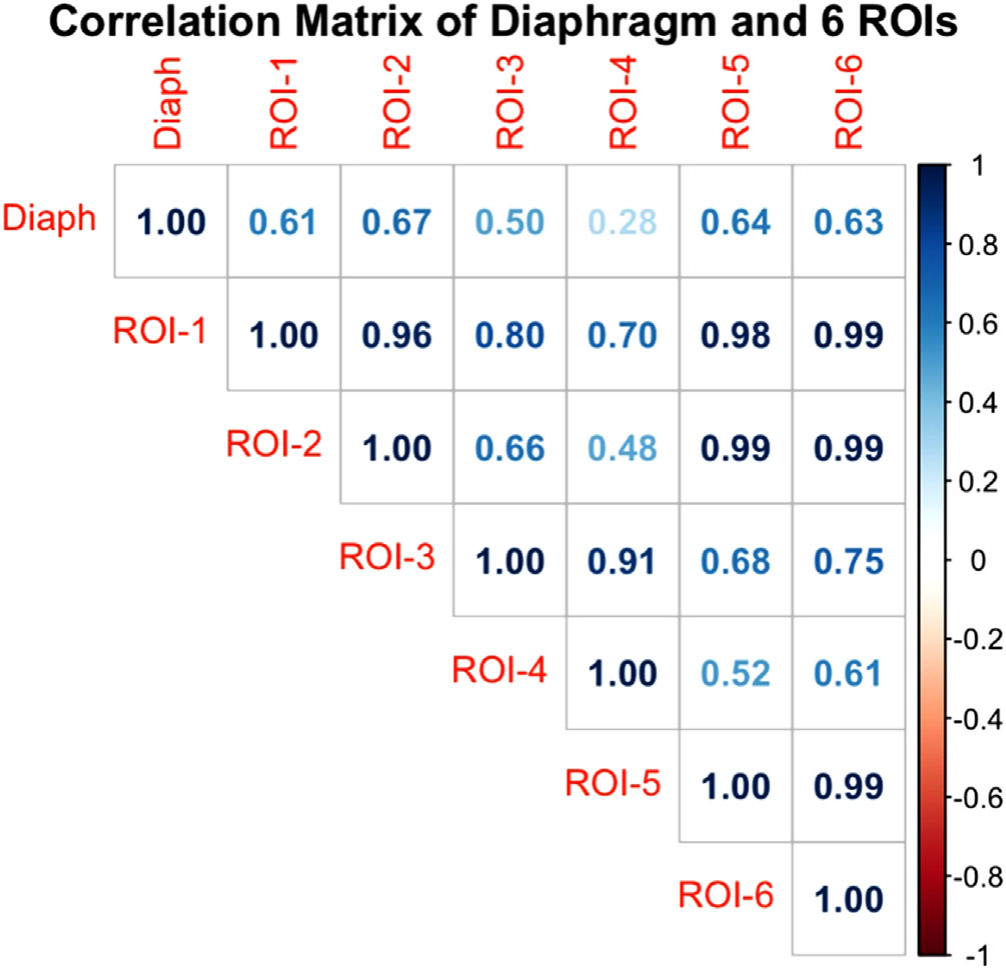
(a) Pearson correlation matrix for six ROIs and the diaphragmatic apex among 20 abdominal patients. The ROI‐2, ROI‐5, and ROI‐6 show the highest correlations with the motion of the diaphragm. (b) Linear regression fitting curves for the six ROIs with the diaphragm motion (in cm) and ROI‐3 and ROI‐4 have the smallest slopes

Table 1 tabulates the mean correlation coefficients of six different ROIs and the slope and R^2^ of their linear regression fitting. These values suggest the capability of the ROIs as respiratory motion surrogates. The ROI‐2, ROI‐5, and ROI‐6 have the highest correlations among all other ROIs, suggesting that these three ROIs can be used clinically to better represent the diaphragm position than the conventional ROIs (ROI‐1, ROI‐3, and ROI‐4). Figure 4 illustrates the correlations and linear regression slopes between the six ROIs and the diaphragm position.

**TABLE 1:** Six regions of interest (ROIs) and their composition of points of interest (POIs), shape, and correlation with the diaphragmatic motion

	Number of POIs				Surrogate of diaphragm		
ROI	Total	Central	Lateral	Simplified topographic shape	Correlation	Slope	*R* ^2^
ROI‐1	12	6	6	□	0.61	0.18	0.37
ROI‐2	8	4 (inferior)	4 (inferior)	□	*0.67*	0.24	0.45
ROI‐3	6	0	6	∇∇	0.50	0.13	0.25
ROI‐4	4	0	4 (superior)	∇∇	0.28	0.06	0.08
ROI‐5	8	6	2 (inferior)	∆	*0.64*	0.24	0.41
ROI‐6	10	6	4 (inferior)	⌂	*0.63*	0.20	0.40

## 4 DISCUSSION

### 4.1 The difference between abdominal ROI and thoracic ROI

As SGRT is becoming more commonplace for breast patient setup (in both FB and DIBH settings), there is a general consensus on what the ROI should be to reproduce the breast position for radiotherapy treatment.^14^, ^17^ For FB setup, the ROI typically includes the central chest area on the sternum since this anatomy is the most stable (more consistent with bony movement and less deformable). Although the breast is often deformable (especially for large patients or patients with pendulous breasts), at least some part of the breast should be included in the ROI to localize the breast for accurate setup. To best reproduce the breast shape and position, a two‐step breast SGRT setup procedure (to align the arm and chin first) has been studied and clinically implemented to minimize breast deformation.^17^ Three different ROI definitions, including B‐ROI (the breast and chest), O‐ROI (the B‐ROI but carving out the central breast area), and inverse T‐ROI (the chest on the sternum and ribs below the breasts), produce similar SGRT breast setup results with only small differences.^14^ For the DIBH setup, as the objective is to monitor the chest wall elevation that allows the heart to separate from the chest wall, so the same ROI can be used to monitor the rise of the thorax from FB to DIBH. Therefore, the ROI definition should be consistent between both FB and DIBH and can be copied from one DICOM surface to the other for preparing SGRT set up in the clinic.

As we move from the thorax to the abdomen to treat liver and pancreatic cancers using DIBH, the ROI definition requires thorough investigation because the clinical goal is to reproduce not only the body position but also the diaphragm position so that the position and shape of the liver and pancreas can be better reproduced for treatment. Therefore, SGRT setup ROI for abdominal DIBH patients would be different from that for FB setup as the DIBH ROI should have the ability to infer the diaphragmatic position using OSI. Thus, the determination of abdominal DIBH ROI should consider the external‐internal motion correlation, which has been intensively studied in the past,^26^, ^27^ but for a different purpose. Almost all previous correlation studies focus on establishing a useful external motion surrogate using a point (RPM), circumference (bellows), or small surface area (OSI) to infer the internal organ/tumor motions. In contrast, the purpose of this study is to seek an optimal ROI for SGRT patient setup in DIBH.

### 4.2 The ROI difference between abdominal FB and DIBH setups

Conventionally, the ROI for FB should be chosen at stable anatomy with minimal motion or deformation, so the surface with rib support would be the best choice. This is because the FB setup aims at aligning patients’ bodies so the FB ROI serves as a surrogate for body positioning. However, when pursuing SGRT for abdominal DIBH setup, the clinical aim is to align the diaphragm, which determines the position and shape of abdominal organs, such as the liver and pancreas, so that the treatment target can be better aligned. Therefore, the DIBH ROI should be an adequate surrogate of the diaphragm position beyond the body position.

In this study, we used 20 abdominal patients’ 4DCT to assess the capability of surface ROI for DIBH by examining the external‐internal correlations at 12 different POIs on the abdominal region and six different ROIs by grouping these POIs differently. It is worthwhile to mention that these 12 POIs are defined relative to patients’ anatomy, and high precision in POI positions are not required, since the POIs are considered as representatives of a small area, rather than a mathematical point, assuming the vicinity surface of the POIs moves similar to the POIs themselves.^18^ Therefore, the correlation analysis among the 20 patients can be used to estimate the correlation of different ROIs using different groups of the POIs. The results have shown that the optimal ROIs with the highest correlation are ROI‐2 (*r* = 0.67), ROI‐5 (*r* = 0.64), and ROI‐6 (*r* = 0.63), on the central and lateral‐inferior abdomen, excluding the superior ribs. The central triangular soft‐tissue ROI without bony support can better represent diaphragm motion, as previously reported.^20^, ^21^ On the contrary, the superior ribs move with the thoracic rib cage, representing more thoracic elevation in the AP direction rather than the diaphragm motion in the SI direction.

In respiratory motion, two major muscles serve as the driving forces: one is the diaphragm that moves soft tissue in the SI direction, causing the abdominal surface to rise and fall, and the other is the intercostal muscles between the ribs that oscillate the chest up and down in the AP direction during respiration. In extreme cases, a patient may be a belly breather or chest breather, in which only one type of muscle group is engaged, causing either abdomen or chest to rise accordingly, as studied before on volunteers.^21^ Moreover, the subject tends to perform deeper inspiration with doubled tidal volumes when putting efforts to do belly or chest breathing, but the external‐internal volumetric relationship persists, suggesting that the correlation is similar between FB and DIBH. More commonly, a patient will use both types of muscles for breathing, so both the chest and abdomen are moving with respiration, although the diaphragm is often used predominantly. Figure 2 shows that the POIs on the mid/inferior abdominal soft tissue have poor correlations with those on the ribs, indicating the motions induced by the two muscle groups behave differently in respiration. When a patient engages in DIBH, other muscles may also be involved, but the diaphragm and intercostal muscles are still the primary muscles to produce DIBH. So, to evaluate the correlation of these abdominal ROIs with the diaphragmatic motion, assessing if and which of the ROIs can serve as the best diaphragmatic motion/positioning surrogates, in addition to serving as body positioning surrogates, 4DCT images of 20 abdominal patients provided sufficient data and clinical evidence for the evaluation. It is worth noting that the same ROI may be used for FB setup or gated setup by using gated OSI acquisition to overcome the breathing motion variation, primarily for AP motion variation, within a breathing cycle. In practice, patients’ diet and daily organ filling may affect the correlation, producing extra uncertainties. So, the patients should be instructed that no large meal within 2 h before their radiotherapy, similar to other abdominal treatments, to reproduce the simulation conditions.

### 4.3 Quality of the abdominal ROIs for DIBH setup

For simplicity, the quality of the 6 ROIs was evaluated based on different sub‐groups of the 12 POIs. The basic assumption that we conducted the study this way was based on the fact that the abdominal areas are relatively flat, and the respiratory‐induced motion is not sensitive to precise POI position so that it can represent a small area at the location. This is directly supported by an AlignRT study, in which small (19 cm^2^), medium (46 cm^2^), and large (178 cm^2^) ROIs produced similar breathing waveforms.^18^ This is also supported by previous AlignRT studies on developing OSI‐based spirometry under an Institutional review board‐approved protocol.^20^, ^21^ In these studies, chest breathers and belly breathers were studied and showed substantial motion differences between the rib‐cage area and abdominal soft‐tissue area,^21^ which was consistent with another OSI observation.^19^ At the same time, gradual changes were observed on the flat surfaces within the rib or soft‐tissue regions. In this study, the correlation of the four superior POIs is greater than 0.9, suggesting their motions are quite synchronized (Figure 2).

From Figures 1, 2, 3 and Table 1, the highest mean external‐internal correlation is 0.7 from the 20 abdominal patients, a common average on external‐internal motion surrogates during FB.^28^ In general, the correlation among patients may not be uniformly high due to both patient‐specific breathing behaviors and the location of the external markers. As discussed above, how a patient engages their muscles during respiration affects the external and internal motions and their synchronization that determines the correlation. For instance, a phase shift may occur that reduces the correlation.^26^, ^28^ On the other hand, the phase shift may also result from the different types (RPM vs. bellows) and locations (near the xiphoid vs. the umbilicus) of the surrogate used for 4DCT scans.^29^, ^30^ When they are placed superiorly near the xiphoid, a phase shift occurs as the RPM represents the soft abdomen while bellows both soft tissue and rib areas, so they sense different motions. When they are placed inferiorly near the umbilicus, both will experience the same abdominal movement outside of the rib cage. In both placement scenarios, these results are consistent with what is observed in this study. As a matter of fact, the 12 POIs can also be used to simulate the RPM and bellows for the external–internal correlation (Figure 1). The RPM is a point‐based surrogate, so each POI may represent the RPM when it is placed at the POI position. As shown in Figure 1, the top row of the triangle graph represents the correlation between the diaphragm and each of the 12 POIs, and clearly, it is location‐dependent: the four most inferior POIs have the highest correlations. The bellows is a circumference‐based surrogate and serves best as a motion surrogate when it is placed close to the biblical point (the four inferior POIs). The RPM/bellows simulations are consistent with the previous findings.^29^, ^30^

The similarity between ROI‐2, ROI‐5, and ROI‐6 suggests that as long as the inferior abdominal soft tissues are predominantly included in the ROI, they would have a similar function as a respiratory surrogate. This observation is consistent with a previous study that showed a high correlation between the bellows and RPM placed adjacently between the biblical point and the bellows,^30^ roughly in the same region of ROI‐2, ROI‐5, and ROI‐6. It is worthwhile to mention that the two most inferior ribs (XI and XII) are the floating ribs, and the costal cartilage in the medial ends of ribs VIII, IX, and X do not connect to the sternum directly, so they are more flexible, somewhat disassociated with the relatively rigid rib cage, and appear to be more responsive to the motion of the diaphragm. This explains why these three ROIs have similar correlations with the diaphragmatic motion. During DIBH, both external and internal organs stop moving, so a possible phase shift in the respiratory waveforms should be no longer present as both diaphragm and surface have reached the DIBH steady state, and timing is no longer relevant. Therefore, without the concern of phase shift, the external–internal correlation is expected to be improved.

### 4.4 Recommendation for clinical ROI for abdominal DIBH treatment

Based on the results of this study and discussion above, ROI‐2, ROI‐5, and ROI‐6 can and should be used as both body positioning and diaphragm positioning surrogates. ROI‐2 and ROI‐6 may have some advantages over ROI‐5 because they cover more lateral body surfaces to provide a unique surface landmark for body alignment. Therefore, ROI‐2 and ROI‐6 can serve as both body positioning and diaphragm positioning surrogates.

For abdominal SGRT DIBH setup, the external body contour from DIBH CT can be used to define the ROI that can be directly used as the DICOM reference for DIBH alignment. Although abdominal SGRT FB setup may be optional for DIBH, it is recommended to be consistent with breast SGRT DIBH setup (FB setup first, followed by DIBH setup), unifying the clinical procedure and workflow, especially for a multi‐center institution.^17^ In the SGRT FB setup, therapists should have ample time to align the patient without asking for DIBH multiple times before treatment. As the DIBH will have very different AP alignment, respiratory‐gated OSI acquisition for FB setup using the same ROI may not be needed, and the same ROI can be used for both FB and DIBH setup in the abdomen, similar to the breast SGRT DIBH setup, facilitating clinical implementation and operation of abdominal SGRT DIBH setup using a uniform consistent setup procedures,^17^ regardless of anatomical sites. The significance of this study is to extend SGRT to serve from a body surface alignment surrogate to an internal organ position surrogate. Therefore, it advances the use of SGRT conceptually in the radiotherapy clinic.

Further study on reproducibility of the diaphragm and abdominal skin surface is warranted in a patient study so that the correlation under DIBH condition can be directly assessed. The CTs in FB and DIBH will be compared and used to assess the correlation under DIBH. The current ROI study has shown the feasibility and validity of evidence‐based ROI for abdominal SGRT DIBH patient setup.

## 5 CONCLUSION

This study investigated and compared several common SGRT ROIs as both a diaphragm and body surrogate for abdominal DIBH setup. The results suggest that the most suitable ROI should cover the central abdominal area and the lateral area inferior to the rib cage. Such an ROI possesses the strongest correlation with the diaphragm positioning while capable of aligning patients’ body positions.

## CONFLICT OF INTEREST

The authors declare that there is no conflict of interest that could be perceived as prejudicing the impartiality of the research reported.

## AUTHOR CONTRIBUTIONS

YS and CZ participated in initial project design process and supervised the students XZ and YL to conduct the data collection and analysis. XZ conducted most data analysis. YL collected most patient data. CZ participated in initial project design process. BM provided funding and supervised the students on this project. GL proposed the project idea, study design, and writing of the manuscript.
